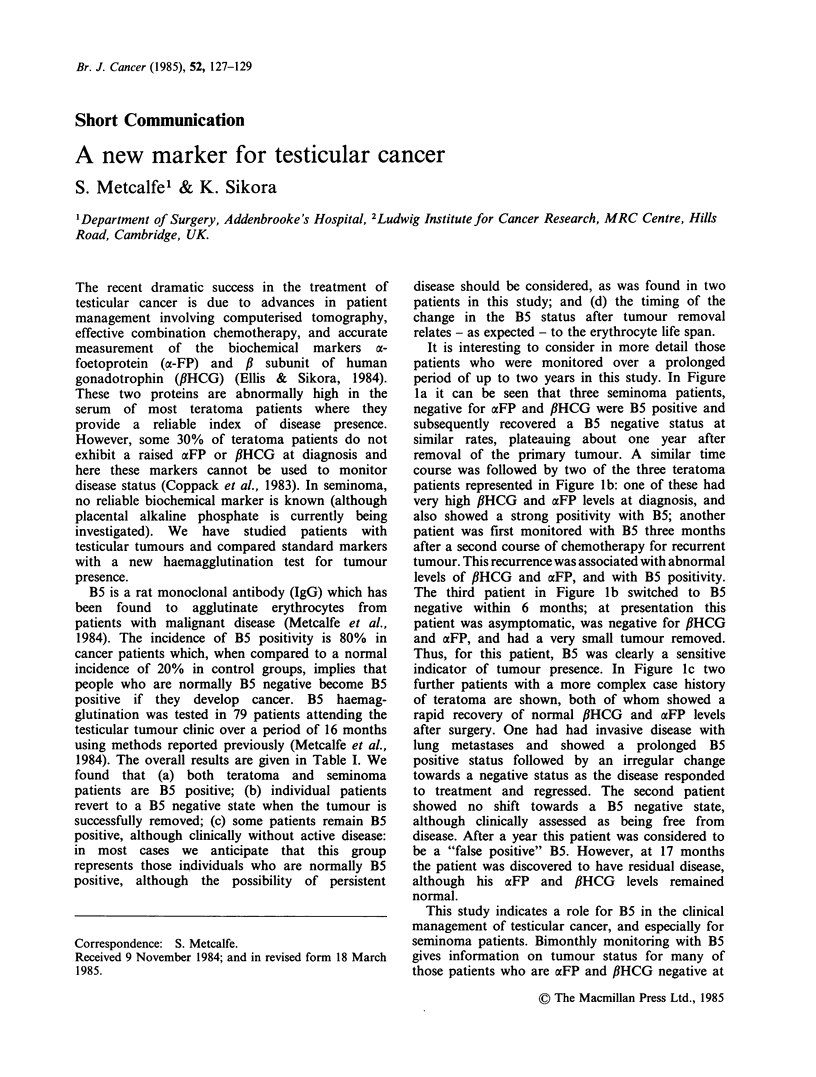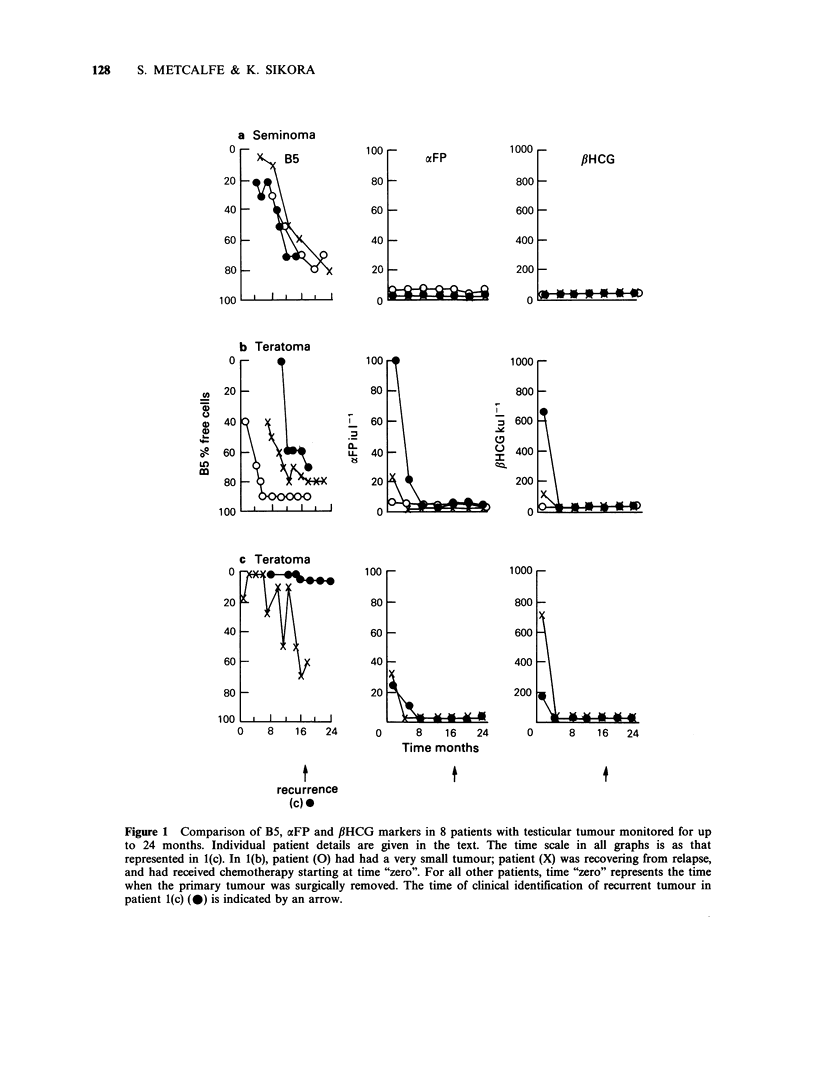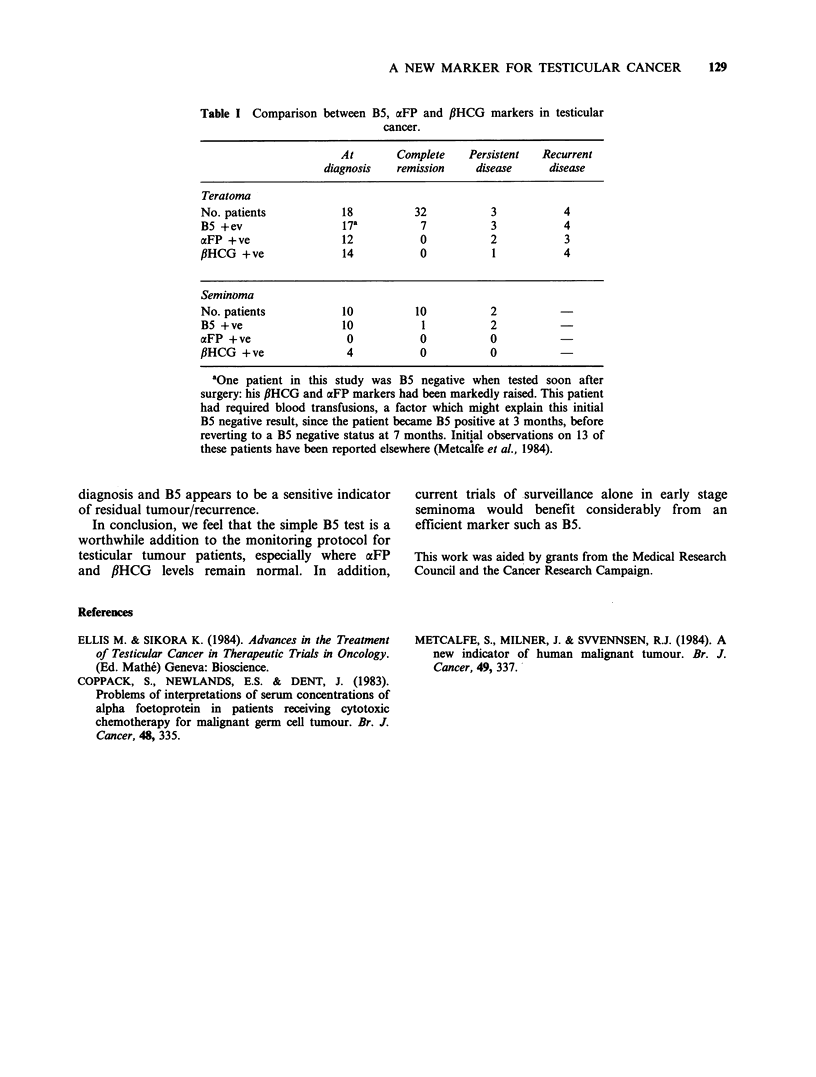# A new marker for testicular cancer.

**DOI:** 10.1038/bjc.1985.160

**Published:** 1985-07

**Authors:** S. Metcalfe, K. Sikora


Br. J. Cancer (1985), 52, 127–129

### Short Communication

### A new marker for testicular cancer

S. Metcalfe[1] & K. Sikora

[1]Department of Surgery, Addenbrooke's Hospital, [2]Ludwig Institute for Cancer Research, MRC Centre, Hills Road, Cambridge, UK.

The recent dramatic success in the treatment of testicular cancer is due to advances in patient management involving computerised tomography, effective combination chemotherapy, and accurate measurement of the biochemical markers α-foetoprotein (α-FP) and β subunit of human gonadotrophin (βHCG) (Ellis & Sikora, 1984). These two proteins are abnormally high in the serum of most teratoma patients where they provide a reliable index of disease presence. However, some 30% of teratoma patients do not exhibit a raised αFP or βHCG at diagnosis and here these markers cannot be used to monitor disease status (Coppack *et al.*, 1983). In seminoma, no reliable biochemical marker is known (although placental alkaline phosphate is currently being investigated). We have studied patients with testicular tumours and compared standard markers with a new haemagglutination test for tumour presence.

B5 is a rat monoclonal antibody (IgG) which has been found to agglutinate erythrocytes from patients with malignant disease (Metcalfe *et al.*, 1984). The incidence of B5 positivity is 80% in cancer patients which, when compared to a normal incidence of 20% in control groups, implies that people who are normally B5 negative become B5 positive if they develop cancer. B5 haemagglutination was tested in 79 patients attending the testicular tumour clinic over a period of 16 months using methods reported previously (Metcalfe *et al.*, 1984). The overall results are given in Table I. We found that (a) both teratoma and seminoma patients are B5 positive; (b) individual patients revert to a B5 negative state when the tumour is successfully removed; (c) some patients remain B5 positive, although clinically without active disease: in most cases we anticipate that this group represents those individuals who are normally B5 positive, although the possibility of persistent disease should be considered, as was found in two patients in this study; and (d) the timing of the change in the B5 status after tumour removal relates – as expected – to the erythrocyte life span.

It is interesting to consider in more detail those patients who were monitored over a prolonged period of up to two years in this study. In Figure 1a it can be seen that three seminoma patients, negative for αFP and βHCG were B5 positive and subsequently recovered a B5 negative status at similar rates, plateauing about one year after removal of the primary tumour. A similar time course was followed by two of the three teratoma patients represented in Figure 1b: one of these had very high βHCG and αFP levels at diagnosis, and also showed a strong positivity with B5; another patient was first monitored with B5 three months after a second course of chemotherapy for recurrent tumour. This recurrence was associated with abnormal levels of βHCG and αFP, and with B5 positivity. The third patient in Figure 1b switched to B5 negative within 6 months; at presentation this patient was asymptomatic, was negative for βHCG and αFP, and had a very small tumour removed. Thus, for this patient, B5 was clearly a sensitive indicator of tumour presence. In Figure 1c two further patients with a more complex case history of teratoma are shown, both of whom showed a rapid recovery of normal βHCG and αFP levels after surgery. One had had invasive disease with lung metastases and showed a prolonged B5 positive status followed by an irregular change towards a negative status as the disease responded to treatment and regressed. The second patient showed no shift towards a B5 negative state, although clinically assessed as being free from disease. After a year this patient was considered to be a "false positive" B5. However, at 17 months the patient was discovered to have residual disease, although his αFP and βHCG levels remained normal.

This study indicates a role for B5 in the clinical management of testicular cancer, and especially for seminoma patients. Bimonthly monitoring with B5 gives information on tumour status for many of those patients who are αFP and βHCG negative at

Correspondence: S. Metcalfe.
Received 9 November 1984; and in revised form 18 March 1985.

© The Macmillan Press Ltd., 1985

**Figure 1** Comparison of B5, αFP and βHCG markers in 8 patients with testicular tumour monitored for up to 24 months. Individual patient details are given in the text. The time scale in all graphs is as that represented in 1(c). In 1(b), patient (O) had had a very small tumour; patient (X) was recovering from relapse, and had received chemotherapy starting at time "zero". For all other patients, time "zero" represents the time when the primary tumour was surgically removed. The time of clinical identification of recurrent tumour in patient 1(c) (●) is indicated by an arrow.

**Table I** Comparison between B5, αFP and βHCG markers in testicular cancer.

| | *At diagnosis* | *Complete remission* | *Persistent disease* | *Recurrent disease* |
|---|---|---|---|---|
| *Teratoma* | | | | |
| No. patients | 18 | 32 | 3 | 4 |
| B5 +ev | 17[a] | 7 | 3 | 4 |
| αFP +ve | 12 | 0 | 2 | 3 |
| βHCG +ve | 14 | 0 | 1 | 4 |
| *Seminoma* | | | | |
| No. patients | 10 | 10 | 2 | — |
| B5 +ve | 10 | 1 | 2 | — |
| αFP +ve | 0 | 0 | 0 | — |
| βHCG +ve | 4 | 0 | 0 | — |

[a]One patient in this study was B5 negative when tested soon after surgery: his βHCG and αFP markers had been markedly raised. This patient had required blood transfusions, a factor which might explain this initial B5 negative result, since the patient became B5 positive at 3 months, before reverting to a B5 negative status at 7 months. Initial observations on 13 of these patients have been reported elsewhere (Metcalfe *et al.*, 1984).

diagnosis and B5 appears to be a sensitive indicator of residual tumour/recurrence.

In conclusion, we feel that the simple B5 test is a worthwhile addition to the monitoring protocol for testicular tumour patients, especially where αFP and βHCG levels remain normal. In addition, current trials of surveillance alone in early stage seminoma would benefit considerably from an efficient marker such as B5.

This work was aided by grants from the Medical Research Council and the Cancer Research Campaign.

### References

ELLIS M. & SIKORA K. (1984). *Advances in the Treatment of Testicular Cancer in Therapeutic Trials in Oncology.* (Ed. Mathé) Geneva: Bioscience.

COPPACK, S., NEWLANDS, E.S. & DENT, J. (1983). Problems of interpretations of serum concentrations of alpha foetoprotein in patients receiving cytotoxic chemotherapy for malignant germ cell tumour. *Br. J. Cancer,* **48,** 335.

METCALFE, S., MILNER, J. & SVVENNSEN, R.J. (1984). A new indicator of human malignant tumour. *Br. J. Cancer,* **49,** 337.